# Spray losses study of two pesticides by UASS in integrated rice–crayfish farming system and acute toxicity evaluation on *Procambarus clarkii*

**DOI:** 10.3389/fpls.2023.1212818

**Published:** 2023-09-11

**Authors:** Yang Liu, Guangyu Wang, Yuanyuan Li, Zhenhua Zhang, Sen Pang, Xiongkui He, Jianli Song

**Affiliations:** ^1^ College of Science, China Agricultural University, Beijing, China; ^2^ College of Agricultural Unmanned Systems, China Agricultural University, Beijing, China; ^3^ College of Plant Protection, China Agricultural University, Beijing, China

**Keywords:** integrated rice-crayfish farming system, unmanned aerial spraying systems, pesticide losses, spray drift, *Procambarus clarkii*, acute toxic effect

## Abstract

**Introduction:**

While the integrated rice-crayfish (*Procambarus clarkii*) farming system (IRCFS) is widely developing in China, the widespread use of Unmanned Aerial Spraying Systems (UASS) to protect rice from pests has led to potential pesticide risk for the crayfish in IRCFS. Therefore, it is crucial to examine UASS’s spray deposition and drift in IRCFS.

**Method:**

In this study, we used the oligonucleotide sequence-tracking / dot-blotting (OSTDB) method to trace pesticide spraying. We collected detailed data not only on spray loss in the paddy fields, but also on spray drift in the breeding ditches caused by upwind and downwind spray areas. Additionally, pesticide residues in the breeding ditches were measured using LC-MS/MS by collecting water samples after pesticide application.

**Results:**

The data analysis indicated that the spray loss in the paddy field was significantly greater than that in the breeding ditches. The spray drift in the breeding ditches, caused by the upwind spray area, was seven times higher than that originating from the downwind spray area. Furthermore, the results also revealed that the bulk flow between the paddy fields and the breeding ditches contributed a substantial amount of pesticide residue to the water body in the breeding ditches. In addition, we investigated the acute toxicities of common insecticides using in paddy fields, including thiamethoxam (THI), chlorantraniliprole (CHI), THI·CHI-Mix and THI·CHI-WG.

**Discussion:**

The results demonstrated that the spray losses and spray drift from UASS spray applications of these pesticides in IRCFS would not cause acute toxicity or death in crayfish. These findings provide important materials for establishing pesticide application standards and guiding the field testing of droplet deposition and drift in IRCFS.

## 1 Introduction

Integrated rice–crayfish farming system (IRCFS), which refers to the simultaneous cultivation of rice and crayfish (*Procambarus clarkii*), has been intensively developed in China due to its extensive benefits (He et al., 2021; Mo et al., 2022; Wu et al., 2022; Yuan et al., 2022; Zhou et al., 2023; Yu et al., 2018a; Yu et al., 2018b). During the rice growing phase, crayfish live harmoniously in the paddy fields that are connected to breeding ditches and migrate back to these ditches during the field drying and rice harvesting periods (Yuan et al., 2021; Chen et al., 2022; Liu et al., 2022). This unique system not only improves the efficiency of water and soil resource utilization (Liu et al., 2022; Zhou et al., 2023; Hou et al., 2021a) but also significantly boosts the economic viability of rice cultivation (Gao et al., 2022; Wu et al., 2022; Xu et al., 2022; Zhang et al., 2022; Hou et al., 2021b).

While IRCFS can mitigate pests and disease (Li et al., 2022; Yu et al., 2022), chemical control remains crucial to prevent losses in rice yield. As wind and drift often carry the droplets to non-target areas (Hu et al., 2022), pesticide application raises environmental concerns since it would pose a potential risk to both the environment and crayfish. The advent of unmanned aerial spraying systems (UASS) has further increased this risk (Wang et al., 2018; Wang et al., 2022). Studies on its impact on IRCFS remain scarce. In IRCFS, breeding ditches hold a higher production value compared to paddy fields. Therefore, it is vital to examine the effects of pesticide spray conditions on IRCFS, such as pesticide residue and its origin in the breeding ditches. This is particularly important in light of the concurrent advancements in new spraying technologies and farming patterns. Compared to traditional water-sensitive paper, which is challenging to use in wet paddy field environments, and dye tracer methods that can easily lead to water field contamination, the new oligonucleotide sequence tracking/dot blotting (OSTDB) technology provides a straightforward way to accurately detect pesticide spray droplets on the surfaces of both targets and non-targets (Song et al., 2021; Zhang et al., 2021). This method will provide detailed information on the deposition distribution and drift of UASS in IRCFS.

With the development of IRCFS, there has been an increasing focus on studies about the acute toxicities of insecticides to non-target crayfish (Biever et al., 2003; Barbee and Stout, 2009; Mamun et al., 2009; Barbee et al., 2010; Yu et al., 2017; Zhu et al., 2022; Liao et al., 2023; Uçkun et al., 2021; Huang et al., 2022; Sun et al., 2022). Thiamethoxam (THI) and chlorantraniliprole (CHI) are two common insecticides that are used in paddy fields and have been widely registered as single-agent products (Mason et al., 2000; Hilton et al., 2016; Wei et al., 2019; Uçkun et al., 2021). In recent years, the blends of THI and CHI have become popular in IRCFS. Therefore, in reference to pesticide residue and its origin in the breeding ditches, it is urgent to evaluate the acute toxicity of mixing THI and CHI to crayfish in IRCFS.

In this study, we developed a risk assessment system to evaluate the impact of pesticide spray droplets on crayfish aquaculture within IRCFS. By employing the dual OSTDB strategy, we traced the origin of losses attributed to pesticide spray drift in both upwind and downwind areas. The pesticide residues of CHI and THI in breeding ditches were also examined. Furthermore, the acute toxicity of mixed THI and CHI to non-target crayfish was evaluated. These results provide crucial data for the safe application of pesticide in IRCFS and to pave the way for its further improvement.

## 2 Materials and methods

### 2.1 Experimental plots

**Figure 1:**
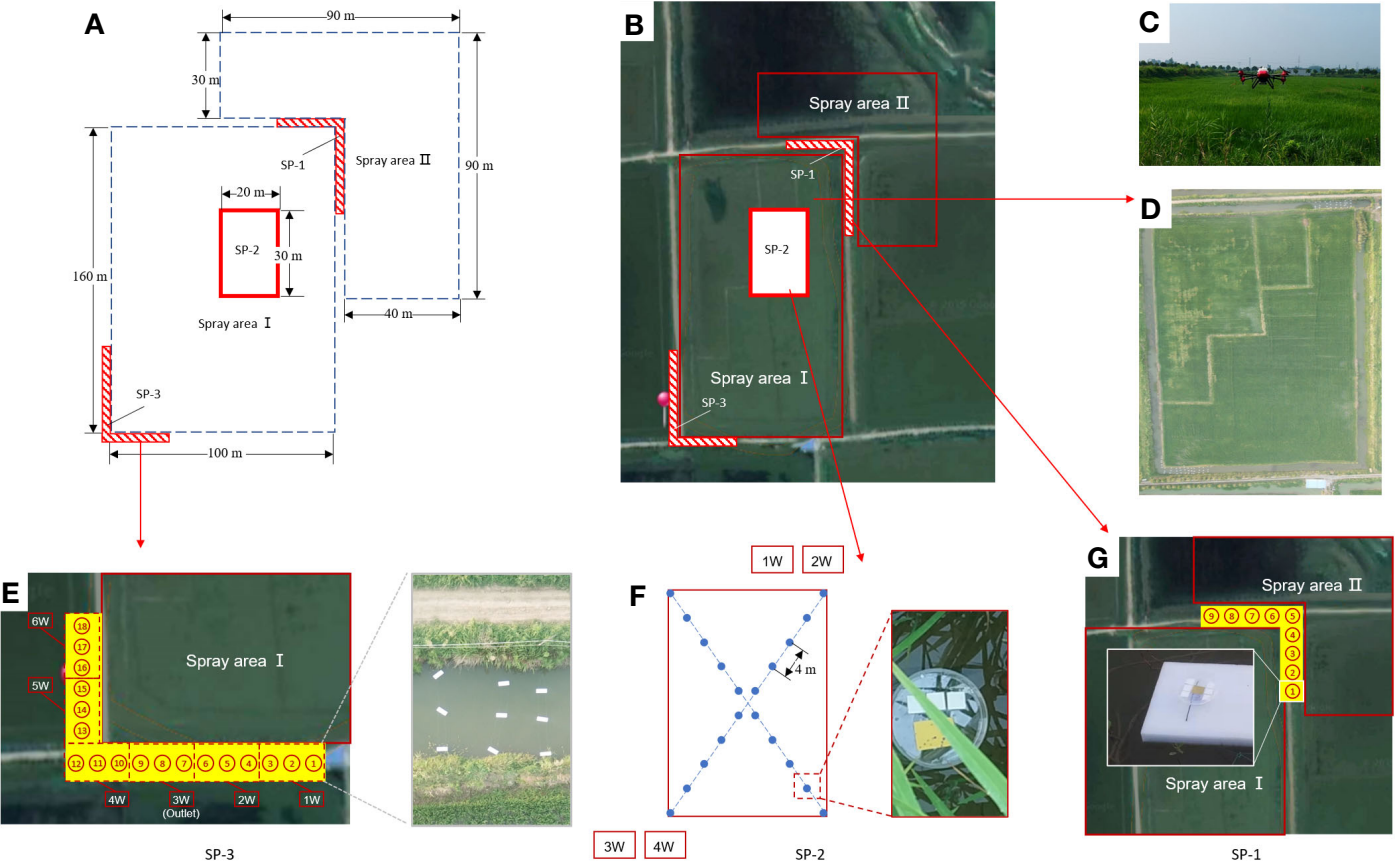
The paddy fields and breeding ditches of integrated rice-crayfish farming system and schematic diagram of the spray areas and the sampling positions. **(A)** Schematic diagram of the division of the spray areas and the sampling positions (SP); **(B)** Distribution of the spray areas and SP; **(C)** The P-20 conducted pesticide application in the experimental field; **(D)** Distribution of paddy fields and breeding ditches; **(E)** Sampling position 3 (SP-3) and on-site diagram of the droplet collectors; **(F)** Sampling position 2 (SP-2) and on-site diagram of the droplet collectors; **(G)** Sampling position 1 (SP-1) and on-site diagram of the droplet collector-NMCs.

The experiment was conducted in July 2019 in Qianjiang City, Hubei Province, China (112°44′ E, 30°17′ N). The test site employed a standard integrated rice–crayfish farming system (**Figure 1D**). A crayfish breeding ditch measuring 4 m in width and 1.5 m in depth was constructed around the paddy field. The marker labels the outlet of the paddy field, which is connected to the breeding ditch. The rice variety used in the experiment was Nanjing 5055, and the species of crayfish was *Procambarus clarkii*. The experiment began 7 days before rice heading. The predominant wind direction at the test site was northeast. In the experiment, a solution of 150 mL/ha of 40% chlorothalonil–thiamethoxam (Virtako, Syngenta, Switzerland) was selected. The solutions were also formulated with 0.1 μmol/L OST-probe-A and OST-probe-B, respectively.

### 2.2 Plant protection unmanned aerial vehicle

A four-rotor electric UASS with a spray tank volume of 10 L (P-20, Guangzhou XAG Technology Co., Ltd.) was used in the experiment for the spray applications in rice–crayfish fields **Figure 1C**. The performance indicators of the UASS are shown in **Table 1**. The P-20 was equipped with high-speed centrifugal atomization nozzles and could implement the spray operation according to an automatically planned route. The spray parameters were set as follows: the flight height was set at 1.7 m above the ground, the spray width was set at 3.0 m, and the spray volume was set at 12 L/ha.

**Table 1:** Main performance indicators of P-20.

Model	Maximum load (L)	Unfold fuselage size (mm × mm × mm)	Operating speed (m/s)	Flight height (m)	Effective spray width (m)	Spray droplet size (μm)
P-20	10	1,852 × 1,828 × 403	2–12	1–30	2–5	90–300

### 2.3 Dual-OST tracer strategy for spray drift assessment

An OSTDB-based tracing technology was used for pesticide mapping of spray droplet and drift. The oligonucleotide sequence tracking probes (OST probe) can achieve specific recognition through complementary base pairing. By mixing the OST probe into the spray tank with pesticides, the droplets containing the OST probe would be collected by nylon membrane collectors (NMCs) modified with complementary probe sequences. Those droplets would then be displayed on the NMCs through a post-processing procedure.

In this experiment, two spraying areas, spray area I (SA-I) and spray area-II (SA-II) were set up, which corresponded to the spray application with liquid containing OST probe-A and OST probe-B, respectively. Nylon membrane collector A (NMC-A) and nylon membrane collector B (NMC-B) were used as the droplet-collecting substrates to detect the spray drift from the two spraying areas on the same position.

### 2.4 Experimental design

The spray-treated area was divided into two parts, SA-I and SA-II, due to the northeast wind direction at the experimental site (**Figure 1B**). SA-I was 100 m × 160 m, while SA-II consisted of two paddy fields with spray areas of 90 m × 30 m and 40 m × 60 m, respectively (**Figure 1A**). A pesticide solution containing OST probe-A was sprayed in SA-I, while a pesticide solution containing OST probe-B was sprayed in SA-II.

Sampling position 1 (SP-1) was situated at the corner between SA-I and SA-II in order to investigate the spray drift caused by both SA-I and SA-II on the breeding ditches. Sampling position 2 (SP-2), located within a 20 m × 30 m area inside SA-I, aimed to detect the distribution of spray losses on the water surface of paddy fields within SA-I. Sampling position 3 (SP-3) was located in the breeding ditches in the downwind direction from SA-I, with the purpose of investigating the spray drift caused by SA-I on the breeding ditch.

#### 2.4.1 Spray drift of the breeding ditch in the upwind of SA-I

In the breeding ditch of SP-1, sampling points were set at intervals of 2 m, with nine sampling points totally in the ditch (**Figure 1G**). At each sampling point, sampling devices fixed on floating boards on the water surface were used to set up three pieces each of NMCs-A and NMCs-B. These were used to trace the spray droplet drift from SA-I and SA-II in the upwind, respectively.

#### 2.4.2 Spray losses in SA-I paddy field

SP-2 was located in the interior region of SA-I paddy field, with 10 sampling points evenly distributed along the diagonal line, each approximately 4 m apart. At each sampling point, sampling devices, which were fixed on floating boards on the water surface, were used to set up three pieces of NMCs-A. These were used to detect the distribution of spray losses on the water surface of SA-I paddy field (**Figure 1F**).

#### 2.4.3 Spray drift of the breeding ditch in the downwind of SA-I

The distribution of sampling points in SP-3 was the same as that in SP-1. As shown in **Figure 1E**, SP-3 was subdivided into 18 locations across six parts. The droplet collection devices were positioned at distances of 0.5, 2.0, and 3.5 m from the boundary of SA-I from SP3-1D to SP3-12D. In addition, six other collection devices were evenly distributed in the east–west ditch. At each sampling point, sampling devices fixed on floating boards on the water surface were used to position three pieces of NMCs-A. These were used to trace the spray droplet drift from SA-I in the downwind.

#### 2.4.4 Water sample collection

Before the pesticide application, blank water samples were collected from the paddy field, with 12 random sampling points selected within the area.

Water samples were also collected from each point in SP-1, SP-2, and SP-3, following the blank water sample collecting procedure, at 1 h after the pesticide application.

At 1, 3, 4, 5, 7,10, 14, and 21 days after the pesticide application, subsequent collections of water samples were made in breeding ditches upwind of the paddy fields (WR-1), in breeding ditches downwind of the paddy fields (WR-2 and WR-3), and in the breeding ditch near the outlet (WR-O). For each water sample, approximately 400 mL was collected and stored at -20°C for analysis.

#### 2.4.5 Measurement of meteorological conditions

A portable weather station “YG-BX” (Chenyun Technology Co., Ltd., China) was employed to monitor the relevant environmental conditions (wind speed, wind direction, temperature, and relative humidity) at a height of 1.0 m over the full duration of the trials. The weather station was positioned 10 m away from the UAV flight path in an open area without any vegetation.

### 2.5 Sample processing for NMCs

The NMC samples were treated using Song’s method (Song et al., 2021). Then, the colored NMCs were scanned at a resolution of 600 dpi, and the grayscale values were measured by using ImageJ. The standard curve representing the relationship between grayscale value and droplet volume was established using droplets formed from the mother liquid of both OST probe-A and OST probe-B. The equation was as follows:


$${\text{G}}_{\text{L}}=k_{0}\times {\text{ V}}_{\text{m}}$$

where $k_{0}$ is the coefficient relating the droplet volume to grayscale value and ${\text{ V}}_{\text{m}}$ is the volume of the OST-probe (μL).

Based on the grayscale values obtained from the samples, the spray drift or droplet loss per unit area was calculated as follows:


$$\beta_{i}={\text{G}}_{Lsmpl}/\left(k_{0}\times {\text{A}}_{\text{col}}\right)$$

where $\beta_{i}$ is the spray drift per unit area (μL/cm^2^), ${\text{G}}_{\text{Lsmpl}}$ is the grayscale value of the sample, and $A_{\text{col}}$ is the collector area (cm^2^).

### 2.6 Sample processing for water samples

In collecting water samples, two separate portions of 15.0 mL were transferred into individual centrifuge tubes. To each tube, 9.0 g of sodium chloride and 15.0 mL of acetonitrile were added. The mixture was then vortexed for 5 min and centrifuged at a speed of 2,800 rpm for another 5 min. From each centrifuge tube, 10 mL of the supernatant was extracted, resulting in a total of 20 mL, which was then transferred into a 50-mL vial. The vial was positioned on a rotary evaporator and evaporated until it reached dryness. Then, 1 mL of acetonitrile was added to the vial to thoroughly rinse the residue from the vial walls. The rinsed solution was filtered through a cellulose acetate filter with a pore size of 0.22 μm and then transferred to a sample vial for the following analysis.

### 2.7 LC–MS/MS analysis

The detection of CHI and THI was referred to the method (Rahul et al., 2020) and analyzed by LC-MS/MS. The test conditions of THI and CHI are shown in **Supplementary Table S1**. The parameters of mass spectrum for THI and CHI are shown in **Supplementary Table S2**.

### 2.8 Determination of LC_50_ values and application

Dose ranges of 3.0–14.0 mg/L of THI and 50.0–120.0 mg/L of CHI as well as 4.0–20.0 mg/L 40% CHI·THI (WG) and 8.0–40.0 mg/L CHI and THI (1:1) mixed dosages were used to determine the 96-h LC_50_ value.

The crayfish were sourced from Longwan Town, Qianjiang City, Hubei Province, China. For this study, glass aquariums with a capacity of 30 L with tubular shelters were used. The studies were conducted at room temperature (23°C ± 1°C) under natural daylight conditions (12-h dark/12-h light). Before applying the pesticide, the crayfish were acclimatized to the laboratory environment for a period of 7 days. Juvenile crayfish were used without regard to their gender. For standardization, crayfish weighing around 5.0 ± 1.0 g and with length of around 5.0 ± 1.0 cm were preferred. Test waters were maintained in the containers with static renewal every 24 h. Six groups were established for each treatment, five of which were exposed to pesticides, while one served as the non-pesticide-applied group (control). Each aquarium housed 10 crayfishes, and the study was replicated three times. Among the live subjects, those who became immobilized over time and exhibited signs of mortality were classified as dead. The count of deceased subjects was recorded at 24-, 48-, 72-, and 96-h intervals. The 96-h LC50 was determined using SPSS 24 probit analysis.

## 3 Results and discussion

### 3.1 Meteorological conditions

**Table 2** shows the meteorological conditions for each spray. The meteorological parameters were in accordance with ISO24253-2 (2015) for field spray droplet deposition test requirements (wind speed, 0–3 m/s; temperature, 10°C–35°C).

**Table 2:** Meteorological parameter records.

Treatment	UV intensity (μW/cm^2^)	Temperature (°C)	Humidity (%)	Wind speed (m/s)	Wind direction (°)
SA-I	3,332.8	34.4	56	1.13	45
SA-II	3,426.5	33.8	57	1.05	38

### 3.2 Pesticide spray drift in breeding ditches upwind of paddy fields

**Figure 2:**
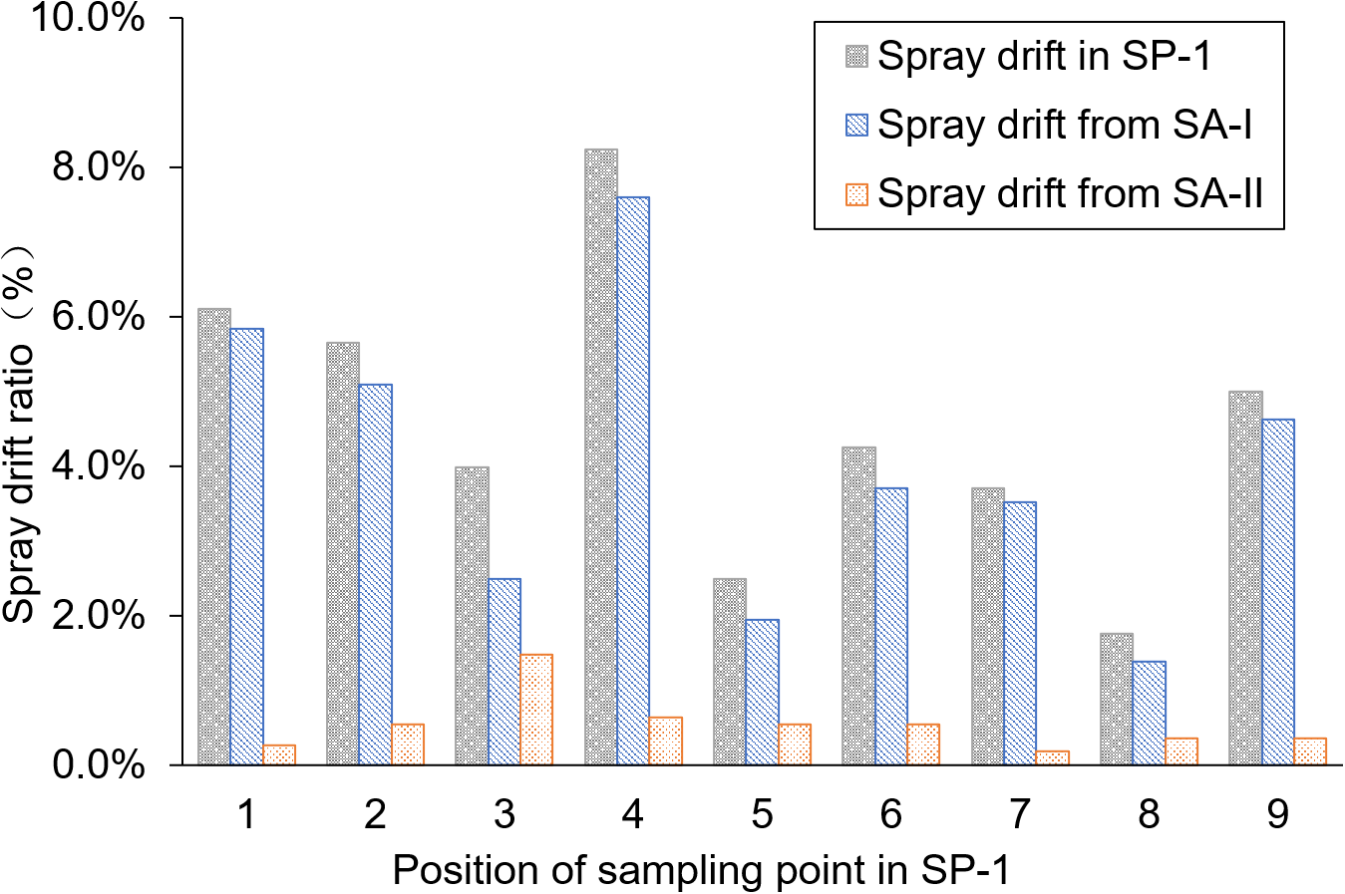
Spray drift distribution in the ditch upwind of the paddy field.

The SP-1 was the breeding ditch located upwind of the SA-I paddy fields, as the wind direction was northeast at the time the pesticide application was conducted. Data on spray drift and pesticide residue in water samples were collected from nine locations (SP-1 to SP1-9). The spray drift ratio of droplets from SA-I and SA-II, obtained by UASS application using the dual-OST-probe tracer method (**Figure 2**), and the total spray drift were analyzed. The results indicated that the main source of spray drift in the breeding ditches upwind of the paddy fields originated from SA-I, which was closer to the ditch.

The average spray drift caused by SA-I was 0.0048 μL/cm^2^, accounting for 4.0% of the theoretical droplet deposition volume (TDDV); the average spray drift caused by SA-II was 0.0007 μL/cm^2^, accounting for 0.6% of TDDV. The total spray drift in the breeding ditches upwind of the paddy fields was 0.0055 μL/cm^2^, accounting for 4.6% of TDDV. The coefficient of variation (CV) of the total spray drift in the breeding ditches upwind of the paddy fields was 0.46, indicating that the distribution of spray drift varies greatly at different locations within the ditch upwind of the paddy fields. This variation may be caused by real-time changes in wind speed during the application process, which alter the distribution of the droplets, or the flight parameters of the UASS may need optimization.

**Figure 3:**
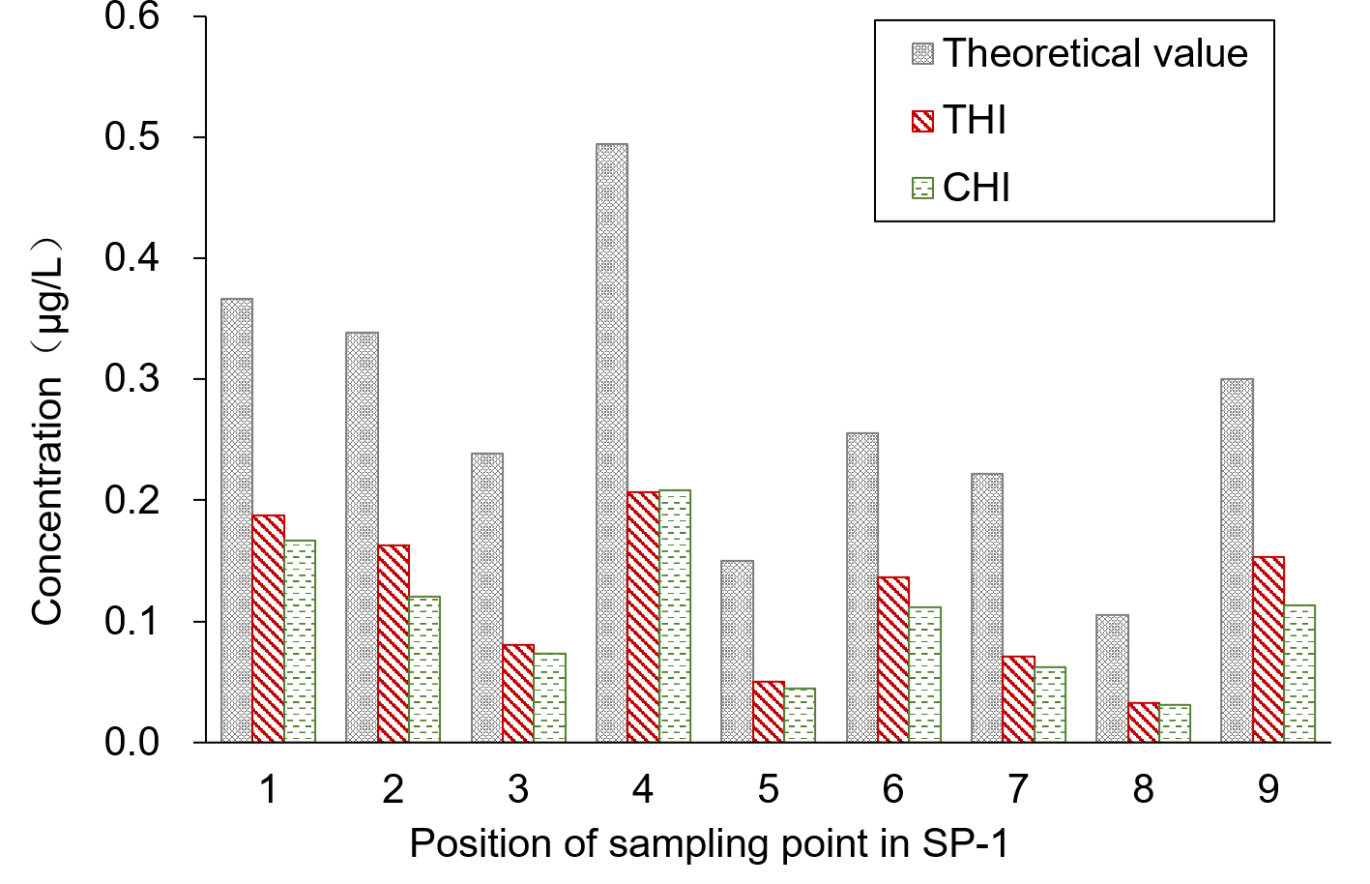
Pesticide residues in the ditch upwind of the paddy field.

The average pesticide residue concentrations of THI and CHI on the water surface of the breeding ditch upwind of the paddy fields, caused by UASS spray application after 1 h, were 0.11 and 0.10 μg/L, respectively. The theoretical concentration of the pesticides was calculated based on the spray drift volume, assuming a diffusion rate of 50 cm/h in the water. As shown in **Figure 3**, this was compared with the pesticide residues detected on the water surface. The pesticide residues measured from the nine locations followed the same trend as the theoretical concentration, but all the data were lower than the theoretical value. The water flow causes the pesticides to move downstream to other areas within the ditch, and the rate of pesticide diffusion in the ditch exceeding the assumed 0.5 m/h may result in a lower proportion of pesticide in the water samples.

### 3.3 Pesticide spray drift in breeding ditches downwind of paddy fields

**Figure 4:**
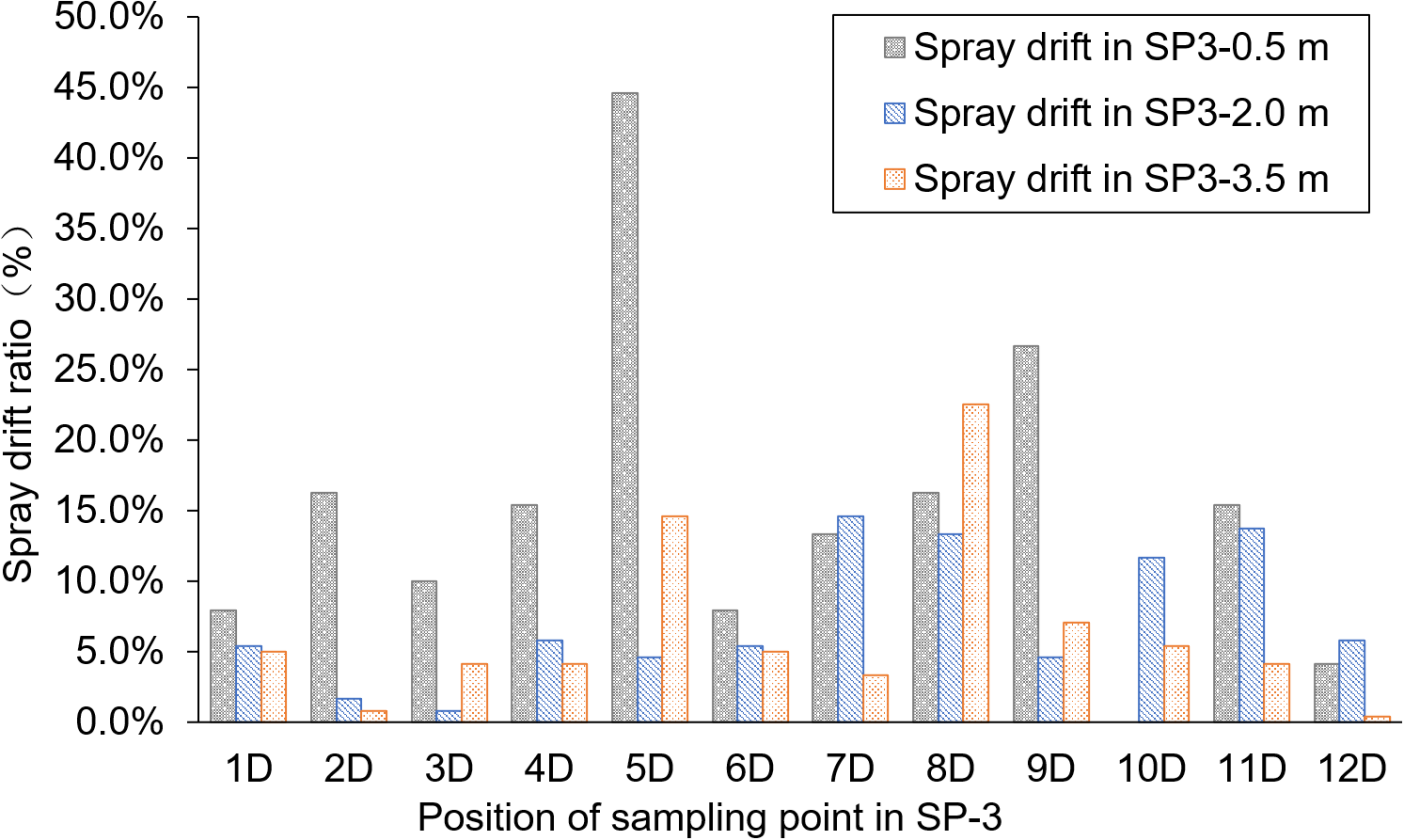
Spray drift distribution in the ditch downwind of the paddy field.

SP-3 was the breeding ditch located downwind of the SA-I paddy fields. Spray droplet drift volume and pesticide residues in water samples were collected from 18 locations in six areas. In this area, the influence of the distance between the breeding ditch and the SA-I on the spray drift in breeding ditches downwind of paddy fields was studied using the OSTDB tracing method. Droplet collection devices were placed at distances of 0.5, 2.0, and 3.5 m from the boundary of SA-I at SP3-1D to SP3-12D. The average spray drift at a distance of 0.5 m from the boundary of SA-I was 0.018 μL/cm^2^, accounting for 14.8% of TDDV with a CV of 0.93. The average spray drift at a distance of 2.0 m from the boundary of SA-I was 0.009 μL/cm^2^, accounting for 7.3% of TDDV with a CV of 0.63. The average spray drift at a distance of 3.5 m from the boundary of SA-I was 0.008 μL/cm^2^, accounting for 6.4% of TDDV with a CV of 0.75 (**Figure 4**). Significant differences were observed in the spray drift between different positions of the breeding ditch and SA-I, which is similar to the distribution pattern observed in the upwind area. The droplet drift at SP3-1D to SP3-12D displayed a wave-like distribution pattern, akin to the spraying swath of the UASS, which may be related to the prolonged hovering time of the drone in the boundary area during flight direction changes.

**Figure 5:**
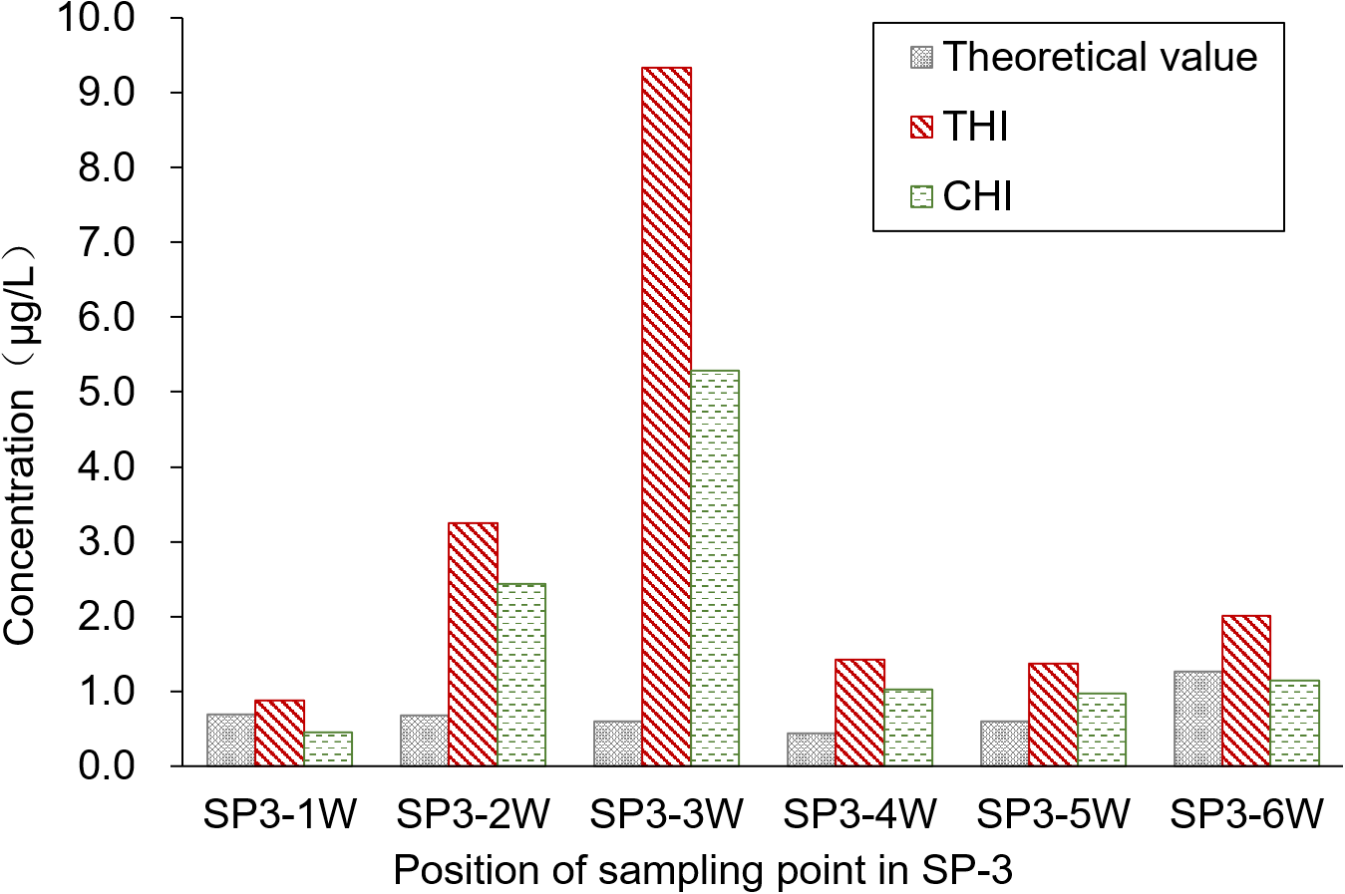
Spray drift distribution in the ditch downwind of the paddy field.

The sampling position was segmented into six water sample collection areas, and the theoretical concentration of the pesticides was calculated based on the spray drift within these areas. **Figure 5** presents a comparison of the pesticide residues detected on the water surface. Contrary to the results from the upwind area, the pesticide concentration detected in the water was higher than the theoretical concentration. The primary reason for this discrepancy is that SP3-3W acts as the outlet connecting the paddy field to the breeding ditch, allowing pesticide losses from the paddy field to flow into the breeding ditch. This flow results in increased pesticide residue in the water. Moreover, the pesticide concentration in the water gradually decreases from the outlet toward both sides. The average concentrations of THI and CHI pesticide residues on the water surface of the breeding ditch downwind of the paddy fields, caused by UASS spray application after 1 h, were 3.05 and 1.88 μg/L, respectively. The significant difference in the residues of THI and CHI could be attributed to the varying solubility of the two pesticides in water. Despite the fact that crayfish are reared in rice paddies during the rice growing period, which could reduce the likelihood of pesticide exposure, there remains a potential risk of contact due to the circulation of water through the outlet.

### 3.4 Pesticide spray losses in paddy fields

In order to study the pesticide spray losses from UAS applications in paddy fields, the OSTDB tracing method was used. Data analysis from 16 sampling points revealed that the distribution of spray droplet losses on the water surface within the paddy field was not uniform. The average spray loss was 0.041 μL/cm^2^, accounting for 34.5% of TDDV with a CV of 0.88 (**Table 3**). The sampling points were located in the water at the bottom of the rice canopy and were randomly distributed. Due to their location, the samplers were covered by the rice canopy. The density of the rice canopy affected the uniformity of spray droplet deposition, leading to differences in the distribution of loss on the water surface. Overall, the average pesticide spray losses were higher than those in breeding ditches. These dissolved pesticides in the water can enter the environment with the water flow, posing potential risks to crayfish.

**Table 3:** Pesticide losses on the water surface of the paddy fields.

Sampling position	Spray losses volume (μL/cm^2^)	Spray losses ratio (%)	Sampling position	Spray losses volume (μL/cm^2^)	Spray losses ratio (%)
2D-11	0.033 ± 0.002	27.2 ± 1.7	2D-21	0.028 ± 0.002	23.1 ± 1.8
2D-12	0.013 ± 0.003	11.1 ± 2.2	2D-22	0.134 ± 0.015	111.4 ± 12.3
2D-13	0.022 ± 0.002	18.3 ± 2.0	2D-23	0.020 ± 0.002	16.4 ± 1.7
2D-14	0.072 ± 0.002	59.7 ± 2.1	2D-24	0.013 ± 0.003	11.1 ± 2.4
2D-15	0.035 ± 0.003	29.4 ± 2.6	2D-25	0.109 ± 0.006	90.6 ± 5.1
2D-16	0.075 ± 0.004	62.8 ± 3.1	2D-26	0.042 ± 0.002	35.3 ± 1.7
2D-17	0.044 ± 0.002	36.7 ± 1.8	2D-27	0.011 ± 0.002	9.4 ± 1.6
2D-18	0.010 ± 0.005	8.0 ± 4.0	2D-28	0.001 ± 0.000	0.6 ± 0.0

The pesticide residues of THI and CHI on the water surface in spraying areas near the upwind boundary (SP2-1W and SP2-2W) and the spraying areas near the downwind boundary (SP2-3W and SP2-4W) in SA-I paddy field were tested 1 h after the pesticide application. As demonstrated in **Table 4**, the pesticide residues in SP2-1W and SP2-2W were higher than those in SP2-3W and SP2-4W. Given that SP2-3W and SP2-4W were closer to the outlet, the rapid flow at this point altered the distribution pattern of pesticide loss in the paddy field. This resulted in discrepancies between the pesticide residues in the water bodies and the theoretical concentration of the pesticides as tested by the OSTDB method.

**Table 4:** Pesticide residues on the water surface of the paddy fields.

Sampling position	Concentration of THI (μg/L)	Concentration of CHI (μg/L)
SP2-1W	2.77 ± 0.12	3.26 ± 0.20
SP2-2W	2.30 ± 003	3.14 ± 0.20
SP2-3W	0.05 ± 0.01	0.81 ± 0.06
SP2-4W	0.18 ± 0.02	0.69 ± 0.07

### 3.5 Pesticide residues in water bodies over time of IRCFS

**Figure 6:**
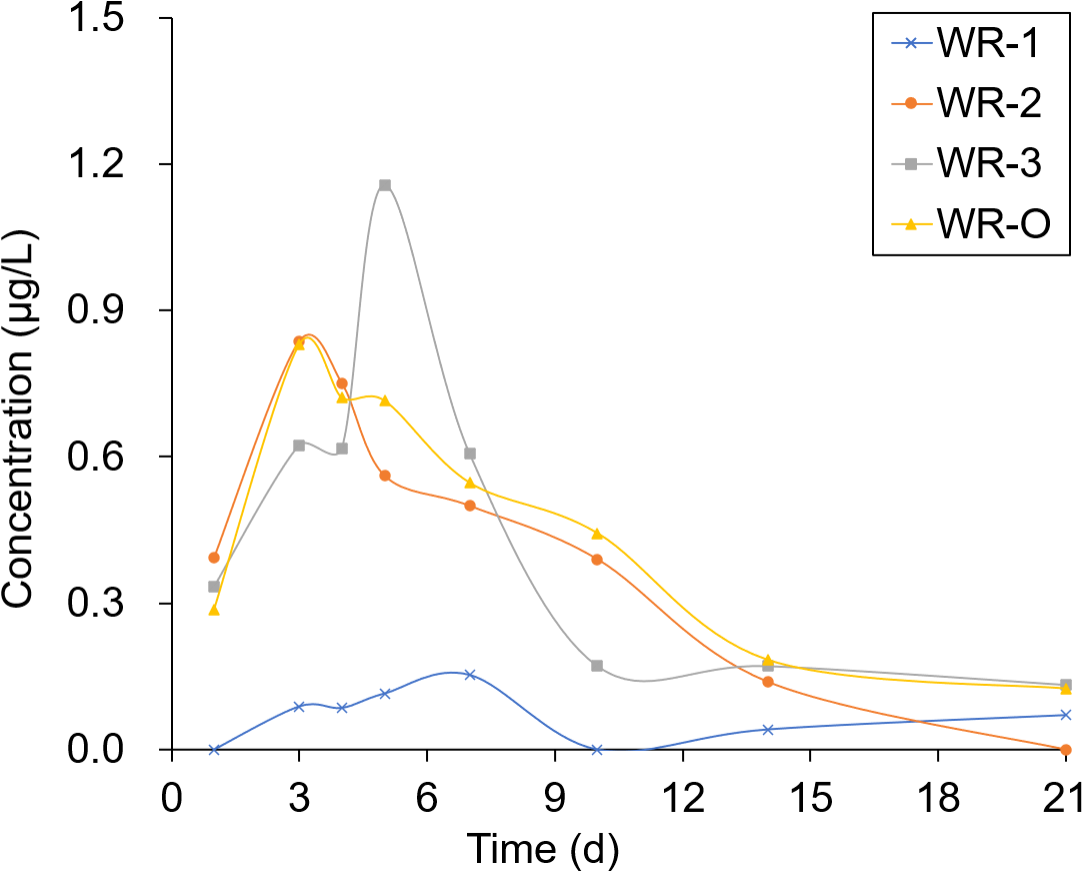
Pesticide residue distribution of THI in the paddy field within 21 days.

**Figure 7:**
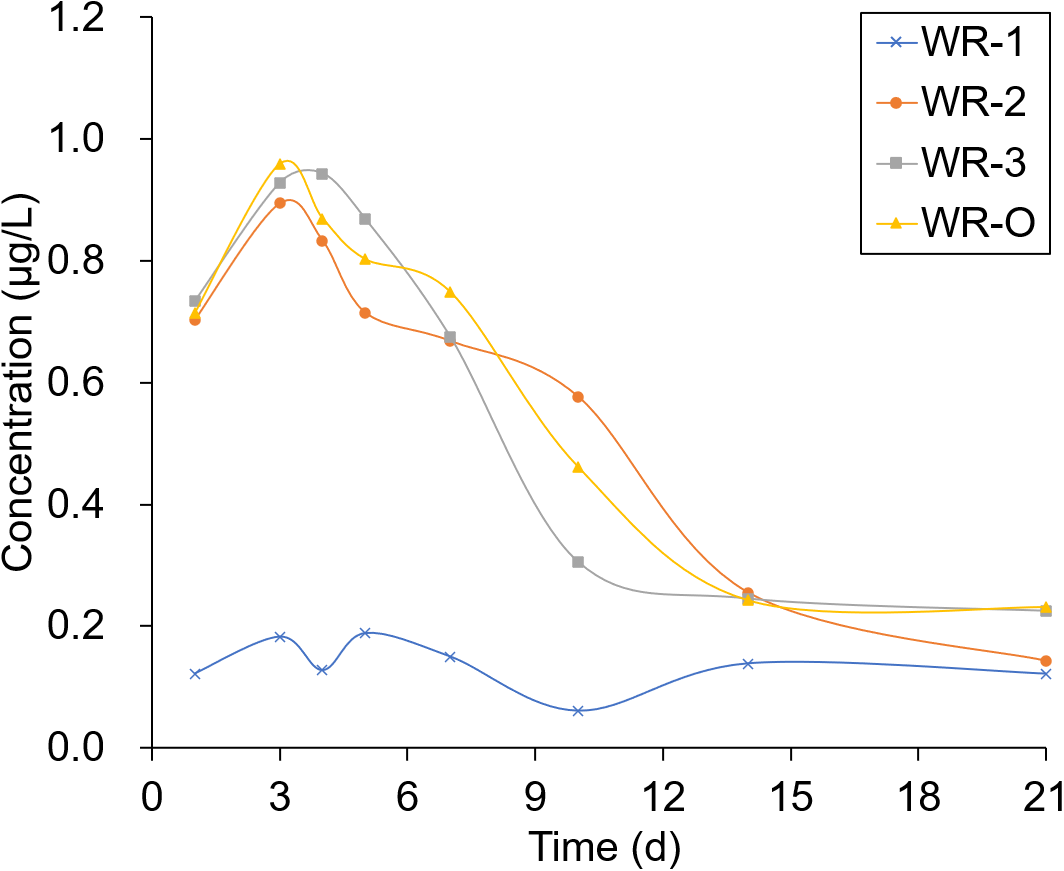
Pesticide residue distribution of CHI in the paddy field within 21 days.

The experimental sites selected for sampling were located in breeding ditches upwind of the paddy fields (WR-1) and in breeding ditches downwind of the paddy fields (WR-2 and WR-3) as well as on the breeding ditch near the outlet (WR-O). Pesticide residues were detected 1, 3, 4, 5, 7, 10, 14, and 21 days after the pesticide application. As shown in **Figures 6**, **7**, the pesticide residue at WR-O, as well as at sampling points WR-2 and WR-3 near the outlet, showed no significant differences. However, the pesticide residue at WR-1 was consistently lower than those in other areas. This can be attributed to the poor mobility of the water body, given that WR-1 is far away from the outlet.

The residues of THI and CHI in the water peaked on the 3rd day, after which they began to gradually decrease. After 21 days of pesticide application, the CHI residues remained detectable at 0.2 μg/L at sampling points located near the outlet. Among the four sampling points, WR-1 exhibited the lowest level of pesticide residue. This is presumably due to its distance from the outlet, limited water mobility, and its upwind location relative to the pesticide application, which aligns with the minimal drift loss observed in the outlet area during the course of the experiment.

### 3.6 Acute toxicity of THI and CHI to crayfish

In order to evaluate the toxic effects of UASS spray application on IRCFS, we further studied the acute toxicity of both THI and CHI as single components and in combination. We conducted a 96-h semi-static acute toxicity test on *P. clarkii* and calculated the lethal concentration that causes 50% mortality (LC_50_). **Table 5** shows the acute toxicity of THI and CHI as single components, with THI demonstrating higher toxicity to juvenile crayfish than CHI. According to the toxicity evaluation criteria of the national standard GB/T 31270.21, the 96-h LC_50_ of THI and CHI on crayfish juveniles was 9.4 and 84.4 mg/L, respectively. This classifies THI as moderately toxic and CHI as less toxic. The relationship between exposure time and toxicity indicated that both THI and CHI exhibited time-dependent effects on the acute lethality of crayfish juveniles, suggesting that the acute toxicity of both insecticides to crayfish juveniles increased with prolonged exposure time.

**Table 5:** Acute toxicity and acute combined toxicity of THI and CHI to *P. clarkii*.

Pesticides	Exposure time (h)	LC_10_ (mg/L)	95% CI (mg/L)	LC_50_ (mg/L)	95% CI (mg/L)	Regression equation	R^2^
THI	24	-	-	-	-	-	-
	48	5.0	1.1–7.1	25.7	15.2–399.9	*y* = 3.1*x* - 4.3	0.963
	72	2.2	0.2–3.9	16.3	10.9–78.8	*y* = 2.5*x* - 3.0	0.952
	96	1.5	0.2–2.9	9.4	7.1–15.7	*y* = 2.8*x* - 2.7	0.992
CHI	24	-	-	-	-	-	-
	48	72.1	14.2–93.1	226.5	141.3–130,122.1	*y* = 4.4*x* - 10.4	0.878
	72	35.8	0.77–54.9	161.4	113.2–3,276.7	*y* = 3.4*x* - 7.4	0.906
	96	32.9	14.3–44.9	84.4	72.3–101.8	*y* = 5.4*x* - 10.3	0.971
CHI·THI·WG	24	-	-	-	-	-	-
	48	4.0	1.4–6.0	15.9	12.7–23.3	*y* = 3.6*x* - 4.4	0.947
	72	2.8	0.9–4.5	11.8	9.2–15.4	*y* = 3.5*x* - 3.8	0.99
	96	2.5	1.0–3.9	8.8	6.8–10.9	*y* = 4.1*x* - 3.8	0.946
THI·CHI·Mix	24	10.7	4.7–14.3	38.0	13.9–47.7	*y* = 4.0*x* - 6.3	0.917
	48	9.2	5.7–11.7	23.2	19.7–29.5	*y* = 5.5*x* - 7.5	0.933
	72	7.6	4.7–9.8	18.7	16.0–22.4	*y* = 5.6*x* - 7.2	0.954
	96	5.8	3.2–7.7	14.6	12.3–17.1	*y* = 5.5*x* - 6.4	0.994

-, the number of deaths was low and did not correlate with concentration to calculate confidence intervals and regression equations.

The acute toxicity of THI and CHI as a 1:1 mixture (THI·CHI-Mix) and 40% THI·CHI water-dispersible granules (THI·CHI-WG) on crayfish is presented in **Table 5**. The toxicity of the THI·CHI-Mix was found to be lower than that of the THI·CHI-WG, with a 96-h LC_50_ of 14.6 mg/L, classifying it as less toxic. Conversely, the 96-h LC_50_ of THI·CHI-WG was 8.8 mg/L, marking it as moderately toxic. The combined toxicity effects were computed using the additivity index method, and the results are presented in **Table 6**. When CHI and THI were mixed in a 1:1 ratio, irrespective of whether it was a mixture of technical materials or a formulated mixture, the combined toxicity effects showed a synergistic action.

**Table 6:** Acute combined effect of THI and CHI to *P. clarkii*.

Pesticides	Exposure time (h)	LC_10_ (mg/L)	S	AI	Joint toxicity evaluation
CHI·THI·WG	48	15.9	0.345	1.902	Synergy
	72	11.8	0.398	1.512	Synergy
	96	8.8	0.522	0.916	Synergy
THI·CHI·Mix	48	23.2	0.502	0.993	Synergy
	72	18.7	0.631	0.584	Synergy
	96	14.6	0.860	0.163	Synergy

The highest concentrations of THI and CHI, detected in the paddy field and the breeding ditches following the UASS spray application, were 9.34 and 5.29 μg/L, respectively. Both of these concentrations were detected at the outlet sampling point. Fortunately, these concentrations were lower than the LC_10_ and LC_50_ values of THI·CHI-WG. Therefore, it is unlikely that the spray losses and spray drift from UASS spray application with these pesticides in IRCFS will cause acute toxicity and death in crayfish (*P. clarkii*).

## 4 Conclusion

In this study, a dual OSTDB method was employed to trace pesticide spray losses in IRCFS. Detailed information regarding the deposition distribution and spray drift of UASS was gathered. The average spray loss in the paddy field accounted for 34.5% of TDDV. The average spray drift in the breeding ditches, caused by upwind SA-I, was 4.0% of the TDDV, while that caused by downwind SA-II was 0.6%. The pesticide residues, measured by LC–MS/MS through the collection of water sample at the same location after pesticide application, displayed the same trend. The data analysis indicated that the spray loss in the paddy field was significantly greater than that in the breeding ditches. The spray drift in the breeding ditches, caused by the upwind spray area, was seven times higher than that originating from the downwind spray area. Furthermore, the results also revealed that the bulk flow between the paddy fields and the breeding ditches contributed a substantial amount of pesticide residue to the water body in the breeding ditches, even more so than the spray drift. A further study about the acute toxicity of THI and CHI to crayfish showed that the 96-h LC_50_ of THI, CHI, THI·CHI-Mix, and THI·CHI-WG on crayfish juveniles was 9.4, 84.4, 14.6, and 8.8 mg/L respectively. The highest concentrations of THI and CHI that were detected in the paddy field and the breeding ditches were lower than that of either THI·CHI-Mix or THI·CHI-WG. These results demonstrated that the spray losses and spray drift from the UASS spray application of these pesticides in IRCFS would not cause acute toxicity or death in crayfish. This interdisciplinary study on both pesticide losses and the acute toxicity of popular THI·CHI blend insecticide in IRCFS provided important materials for the establishment of pesticide application standards and guiding the field testing of droplet deposition and drift in IRCFS.

## Data availability statement

The raw data supporting the conclusions of this article will be made available by the authors, without undue reservation.

## Author contributions

JS and ZZ contributed to the conception of the study. YaL, JS, XH, and ZZ designed and directed the experiments. YaL and GW conducted the indoor experiments. YaL, GW, YuL, and SP performed the field trials. YaL and GW contributed significantly to analysis and manuscript preparation. YaL performed the data analyses and wrote the manuscript. YaL, SP, JS, XH, and ZZ helped perform the analysis with constructive discussions. All authors contributed to the article and approved the submitted version.

## References

[B1] Barbee G. C. McClain W. R. Lanka S. K. Stout M. J. (2010). Acute toxicity of chlorantraniliprole to non-target crayfish (Procambarus clarkii) associated with rice–crayfish cropping systems. Pest Manage. Sci. 66 (9), 996–1001. doi: 10.1002/ps.1972 20730992

[B2] Barbee G. C. Stout M. J. (2009). Comparative acute toxicity of neonicotinoid and pyrethroid insecticides to non-target crayfish (Procambarus clarkii) associated with rice-crayfish crop rotations. Pest Manage. Sci. 65 (11), 1250–1256. doi: 10.1002/ps.1817 19623546

[B3] Biever R. C. Hoberg J. R. Jacobson B. Dionne E. Sulaiman M. McCahon P. (2003). ICON® rice seed treatment toxicity to crayfish (Procambarus clarkii) in experimental rice paddies. Environ. Toxicol. Chem. 22 (1), 167–174. doi: 10.1002/etc.5620220122 12503761

[B4] Chen L. Xu J. Wan W. Xu Z. Hu R. Zhang Y.. (2022). The microbiome structure of a rice-crayfish integrated breeding model and its association with crayfish growth and water quality. Microbiol. Spectr. 10 (2), e02204–e02221. doi: 10.1128/spectrum.02204-21 35384719 PMC9045173

[B5] Gao H. Li Y. Zhou Y. Guo H. Chen L. Yang Q.. (2022). Influence of mechanical transplanting methods and planting geometry on grain yield and lodging resistance of indica rice taoyouxiangzhan under rice–crayfish rotation system. Agron 12 (5), 1029. doi: 10.3390/agronomy12051029

[B6] He M. Liu F. Wang F. (2021). Quantitative analysis of density dependent resource utilization, cannibalism, and competition of the red swamp crayfish (Procambarus clarkii) in rice-crayfish cocultures without supplementary food. Aquaculture 543, 736966. doi: 10.1016/j.aquaculture.2021.736966

[B7] Hilton M. J. Jarvis T. D. Ricketts D. C. (2016). The degradation rate of thiamethoxam in european field studies. Pest Manage. Sci. 72 (2), 388–397. doi: 10.1002/ps.4024 25884469

[B8] Hou J. Styles D. Cao Y. Ye X. (2021a). The sustainability of rice-crayfish coculture systems: a mini review of evidence from Jianghan plain in China. J. Sci. Food Agr 101 (9), 3843–3853. doi: 10.1002/jsfa.11019 33336495

[B9] Hou J. Wang X. Xu Q. Cao Y. Zhang D. Zhu J. (2021b). Rice-crayfish systems are not a panacea for sustaining cleaner food production. Environ. Sci. pollut. R. 28, 22913–22926. doi: 10.1007/s11356-021-12345-7 33432412

[B10] Hu P. Zhang R. Yang J. Chen L. (2022). Development status and key technologies of plant protection UAVs in China: a review. Drones. 6 (11), 354. doi: 10.3390/drones6110354

[B11] Huang X. Li M. Huang Y. Yang H. Geng Y. Ouyang P.. (2022). Microbiome analysis reveals microecological advantages of emerging ditchless rice-crayfish co-culture mode. Front. Microbiol. 13, 892026. doi: 10.3389/fmicb.2022.892026 35935240 PMC9355531

[B12] ISO24253-2 (2015). Crop protection equipment - Spray deposition test for field crop - Part 2: Measurement in a crop. ISO Central Secretariat: International Organization for Standardization.

[B13] Li C. Huang L. Zhang Y. Guo X. Cao N. Yao C.. (2022). Effects of triazole plant growth regulators on molting mechanism in Chinese mitten crab (*Eriocheir sinensis*). Fish Shellfish Immunol. 131, 646–653. doi: 10.1016/j.fsi.2022.10.059 36330873

[B14] Liao M. Liang Z. Wu R. Xiao J. Gao Q. Cao H. (2023). Residue behavior of cyantraniliprole and its ecological effects on Procambarus clarkii associated with the rice–crayfish integrated system. Pest Manage. Sci. 79 (5), 1868–1875. doi: 10.1002/ps.7364 36654512

[B15] Liu T. Li C. Tan W. Wang J. Feng J. Hu Q.. (2022). Rice-crayfish co-culture reduces ammonia volatilization and increases rice nitrogen uptake in central China. Agr. Ecosyst. Environ. 330, 107869. doi: 10.1016/j.agee.2022.107869

[B16] Mamun M. I. R. Park J. H. Choi J. H. Kim H. K. Choi W. J. Han S. S.. (2009). Development and validation of a multiresidue method for determination of 82 pesticides in water using GC. J. Sep. Sci. 32 (4), 559–574. doi: 10.1002/jssc.200800606 19212978

[B17] Mason G. Rancati M. Bosco D. (2000). The effect of thiamethoxam, a second-generation neonicotinoid insecticide, in preventing transmission of tomato yellow leaf curl geminivirus (TYLCV) by the whitefly *Bemisia tabaci* (Gennadius). Crop Prot. 19 (7), 473–479. doi: 10.1016/S0261-2194(00)00042-9

[B18] Mo A. Dang Y. Wang J. Liu C. Yang H. Zhai Y.. (2022). Heavy metal residues, releases and food health risks between the two main crayfish culturing models: Rice-crayfish coculture system versus crayfish intensive culture system. Environ. pollut. 305, 119216. doi: 10.1016/j.envpol.2022.119216 35395351

[B19] Rahul C. Harischandra R. Pallavi S. Rachappa V. Pramesh D. Bheemanna M. (2020). LC-ESI-MS/MS method for determination of chlorantraniliprole residue and its dissipation kinetics in pigeonpea. Pest. Res. J. 32 (1), 96–106. doi: 10.5958/2249-524X.2020.00013.8

[B20] Song J. Liu Y. He X. Zhang Z. Pang S. Xu S.. (2021). Kit and method for simultaneously detecting droplet drift or deposition of multiple sprays, U.S. Patent US20210214777A1 (Washington, DC: U.S. Patent and Trademark Office).

[B21] Sun Q. Khoshnevisan. B. Zhu J. Wang W. Liu Y. Pan J.. (2022). Comprehensive assessment of integrated rice-crayfish farming system as a new paradigm to air-water-food nexus sustainability. J. Clean. Prod. 377, 134247. doi: 10.1016/j.jclepro.2022.134247

[B22] Uçkun M. Yoloğlu E. Uçkun A. A. Öz Ö.B. (2021). Acute toxicity of insecticide thiamethoxam to crayfish (Astacus leptodactylus): alterations in oxidative stress markers, ATPases and cholinesterase. Acta Chim. Slov 68 (3), 521–531. doi: 10.17344/acsi.2021.6823 34897546

[B23] Wang X. He X. Song J. Wang Z. Wang C. Wang S.. (2018). Drift potential of uav with adjuvants in aerial applications. Int. J. Agric. Biol. Eng. 11 (5), 54–58. doi: 10.25165/j.ijabe.20181105.3185

[B24] Wang C. Liu Y. Zhang Z. Han L. Li Y. Zhang H.. (2022). Spray performance evaluation of a six-rotor unmanned aerial vehicle sprayer for pesticide application using an orchard operation mode in apple orchards. Pest Manage. Sci. 6, 78. doi: 10.1002/ps.6875 35306733

[B25] Wei Y. Yan R. Zhou Q. Qiao L. Zhu G. Chen M. (2019). Monitoring and mechanisms of chlorantraniliprole resistance in *Chilo suppressalis* (Lepidoptera: crambidae) in China. J. Econ Entomol. 112 (3), 1348–1353. doi: 10.1093/jee/toz001 30715398

[B26] Wu Y. Li Y. Niu L. Zhang W. Wang L. Zhang H. (2022). Nutrient status of integrated rice-crayfish system impacts the microbial nitrogen-transformation processes in paddy fields and rice yields. Sci. Total Environ. 836, 155706. doi: 10.1016/j.scitotenv.2022.155706 35526617

[B27] Xu Q. Peng X. Guo H. Che Y. Dou Z. Xing Z.. (2022). Rice-crayfish coculture delivers more nutrition at a lower environmental cost. Sustain. Prod. Consump. 29, 14–24. doi: 10.1016/j.spc.2021.09.020

[B28] Yu L. Li C. Zhang Y. Guo X. Cao N. Guo S.. (2022). Residue monitoring of propiconazole in the rice crab co-culture field and its toxicity and bioaccumulation to eriocheir sinensis. Front. Environ. Sci. 10, 848348. doi: 10.3389/fenvs.2022.848348

[B29] Yu J. Ren Y. Xu T. Li W. Xiong M. Zhang T.. (2018a). Physicochemical water quality parameters in typical rice-crayfish integrated systems (RCIS) in China. Int. J. Agr. Biol. Eng. 11 (3), 54–60. doi: 10.25165/j.ijabe.20181103.3761

[B30] Yu J. Xu E. G. Li W. Jin S. Yuan T. Liu J.. (2018b). Acute toxicity of an emerging insecticide pymetrozine to Procambarus clarkii associated with rice-crayfish culture (RCIS). Int. J. Environ. R. Pub. He 15 (5), 984. doi: 10.3390/ijerph15050984 PMC5982023 29757963

[B31] Yu J. Xu E. G. Ren Y. Jin S. Zhang T. Liu J.. (2017). Mixture toxicity of bensulfuron-methyl and acetochlor to red swamp crayfish (Procambarus clarkii): Behavioral, morphological and histological effects. Int. J. Environ. R. Pub. He 14 (12), 1466. doi: 10.3390/ijerph14121466 PMC5750885 29186931

[B32] Yuan P. L. Wang J. P. Can G. U. O. Guo Z. Y. Yao G. U. O. Cao C. G. (2022). Sustainability of the rice–crayfish farming model in waterlogged land: A case study in Qianjiang County, Hubei Province, China. J. Integr. Agr 21 (4), 1203–1214. doi: 10.1016/S2095-3119(21)63787-5

[B33] Yuan P. Wang J. Chen S. Guo Y. Cao C. (2021). Certified rice–crayfish as an alternative farming modality in waterlogged land in the Jianghan Plain region of China. Agron. J. 113 (6), 4568–4580. doi: 10.1002/agj2.20694

[B34] Zhang Z. Du L. Xiao Z. Li C. Wang Z. Zhou P.. (2022). Rice-crayfish farming increases soil organic carbon. Agr. Ecosyst. Environ. 329, 107857. doi: 10.1016/j.agee.2022.107857

[B35] Zhang Z. Li Z. Wu X. Song J. Liu Y. Zhu L.. (2021). Kit and method for detecting droplet drift or deposition characteristics of spray, U.S. Patent US17/214,739 (Washington, DC: U.S. Patent and Trademark Office).

[B36] Zhou Y. Harrison M. T. Liu K. Xiao L. Zhu J. Wang M.. (2023). Field distribution characteristics and influencing factors of crayfish in rice-crayfish integrative system. Aquaculture 571, 739456. doi: 10.1016/j.aquaculture.2023.739456

[B37] Zhu X. Ji L. Cheng M. Wei H. Wang Z. Ning K. (2022). Sustainability of the rice-crayfish co-culture aquaculture model: microbiome profiles based on multi-kingdom analyses. Environ. Microbiome. 17, 27. doi: 10.1186/s40793-022-00422-4 35599327 PMC9124410

