# Supplementary material for: Spray losses study of two pesticides by UASS in integrated rice–crayfish farming system and acute toxicity evaluation on Procambarus clarkii
Source: Front Plant Sci. 2023 Sep 11;14:1212818. doi: 10.3389/fpls.2023.1212818 (PMC10519795; doi:10.3389/fpls.2023.1212818)
Supplement: Supplementary file 1 [file Presentation_1.pdf]

Supplementary Material

Spray losses study of two pesticides by UASS in integrated rice-crayfish farming system and acute toxicity evaluation on *Procambarus clarkii*

Yang Liu[1,2], Guangyu Wang[1], Yuanyuan Li[3], Zhenhua Zhang[1,2*], Sen Pang[1], Xiongku He[1,2], Jianli Song[1,2*]

[1]College of Science, China Agricultural University, Beijing, China

[2]College of Agricultural Unmanned Systems, China Agricultural University, Beijing, China

[3]College of plant protection, China Agricultural University, Beijing, China

*** Correspondence:**
Corresponding Author 1: Jianli Song songjianli@cau.edu.cn

Corresponding Author 2: Zhenhua Zhang zhangzhh@cau.edu.cn

Supplementary Table 1 Test conditions of thiamethoxam and chlorantraniliprole

LC-MS/MS conditions	
Chromatographic Columns	Athena C_{18} WP 3.0 μm×2.1 mm×50 mm
liquid phase	ACN: FA (0.1%, v/v) = 85:15
Flow rate	0.2 mL/min
Column temperature	35 °C
Injection volume	5 μL
Scanning mode	MRM
Ionization mode	ESI (+)
Dry gas	N_2
Nebulizer Pressure	35 PSI
Drying gas flow rate	11.0 L/min
Drying gas temperature	300 °C
Capillary Voltage	4.0 kV

Supplementary Table 2 The parameters of mass spectrum for thiamethoxam and chlorantraniliprole

Pesticides	Retention time (min)	Quantitative ion pair (m/z)	Qualitative ion pair (m/z)	Collision energy (eV)	Fragmentation voltage (V)
Thiamethoxam	0.85	292->211	292->211	10	110
			292->132	20	
Chlorantraniliprole	1.13	484->286	484->286	10	110
			484->453	20	

Supplementary Table 3 Pesticide droplet losses on water surface of paddy fields

Sampling position	Spray losses volume (μL/cm^2)			Spray losses ratio (%)		
	Repeat-1	Repeat-2	Repeat-3	Repeat-1	Repeat-2	Repeat-3
2D-11	0.033	0.035	0.030	27.5%	29.2%	25.0%
2D-12	0.011	0.012	0.017	9.2%	10.0%	14.2%
2D-13	0.019	0.022	0.025	15.8%	18.3%	20.8%
2D-14	0.069	0.075	0.071	57.5%	62.5%	59.2%
2D-15	0.031	0.037	0.038	25.8%	30.8%	31.7%
2D-16	0.075	0.071	0.080	62.5%	59.2%	66.7%
2D-17	0.041	0.045	0.046	34.2%	37.5%	38.3%
2D-18	0.003	0.013	0.013	2.4%	10.8%	10.8%
2D-21	0.025	0.029	0.030	20.8%	24.2%	25.0%

2D-22	0.132	0.153	0.117	110.0%	127.5%	97.5%
2D-23	0.022	0.017	0.020	18.3%	14.2%	16.7%
2D-24	0.010	0.017	0.013	8.3%	14.2%	10.8%
2D-25	0.102	0.108	0.117	85.0%	90.0%	97.5%
2D-26	0.042	0.045	0.040	35.0%	37.5%	33.3%
2D-27	0.010	0.010	0.014	8.3%	8.3%	11.7%
2D-28	0.001	0.001	0.001	0.8%	0.8%	0.8%

Supplementary Table 4 Pesticide residues on water surface of paddy fields

Sampling position	Concentration of THI (μg/L)			Concentration of CHI (μg/L)		
	Repeat-1	Repeat-2	Repeat-3	Repeat-1	Repeat-2	Repeat-3
SP2-1W	2.71	2.94	2.66	3.20	3.53	3.06
SP2-2W	2.30	2.33	2.27	3.22	2.86	3.32
SP2-3W	0.03	0.06	0.04	0.90	0.75	0.79
SP2-4W	0.20	0.19	0.16	0.77	0.60	0.70

Supplementary Table 5 Deaths of *P. clarkii* with THI treatment.

C_{THI}* (mg/L)	24h			48h			72h			96h		
	R1**	R2	R3	R1	R2	R3	R1	R2	R1	R2	R3	R1
0.0	0	0	0	0	0	0	0	0	0	0	0	0
3.0	1	0	0	2	0	0	3	0	1	3	1	2
5.0	0	1	0	0	2	1	0	4	2	3	3	4
7.0	0	0	0	1	1	2	2	4	4	3	5	4
10.0	0	1	1	1	1	4	3	2	5	4	5	6
14.0	2	0	1	4	0	6	5	2	7	6	6	7

* C_{THI}, concentration of Thiamethoxam, mg/L
** R1, R2 and R3 represent for repeat 1, repeat 2 and repeat 3, respectively.

Supplementary Table 6 Deaths of *P. clarkii* with CHI treatment.

C_{CHI}* (mg/L)	24h			48h			72h			96h		
	R1**	R2	R3	R1	R2	R3	R1	R2	R1	R2	R3	R1
0.0	0	0	0	0	0	0	0	0	0	0	0	0
50.0	0	0	0	0	1	0	1	2	1	3	3	1
60.0	0	1	1	0	1	2	2	2	3	2	3	5
80.0	1	1	0	2	1	1	2	2	3	3	4	6
100.0	1	0	1	1	2	2	3	4	3	6	5	6
120.0	1	2	1	3	2	2	5	3	4	8	6	8

* C_{CHI}, concentration of Chlorantraniliprole, mg/L
** R1, R2 and R3 represent for repeat 1, repeat 2 and repeat 3, respectively.

Supplementary Table 7 Deaths of *P. clarkii* with THI·CHI·WG treatment.

C_{TCW}* (mg/L)	24h			48h			72h			96h		
	R1**	R2	R3	R1	R2	R3	R1	R2	R1	R2	R3	R1
0.0	0	0	0	0	0	0	0	0	0	0	0	0
4.0	0	0	0	2	1	1	2	2	1	3	2	2
8.0	1	1	0	2	3	1	3	5	3	3	6	4
12.0	1	1	1	4	5	2	6	5	3	6	6	5
16.0	1	2	1	5	6	5	6	6	7	7	6	9
20.0	3	0	1	8	5	5	8	6	7	9	8	8

* C_{TCW}, concentration of THI·CHI·WG, mg/L

** R1, R2 and R3 represent for repeat 1, repeat 2 and repeat 3, respectively.

Supplementary Table 8 Deaths of *P. clarkii* with THI·CHI·Mix treatment.

C_{TCM}* (mg/L)	24h			48h			72h			96h		
	R1**	R2	R3	R1	R2	R3	R1	R2	R1	R2	R3	R1
0.0	0	0	0	0	0	0	0	0	0	0	0	0
8.0	0	0	1	0	0	1	0	1	1	2	2	2
11.2	1	0	2	1	1	3	2	1	5	3	2	6
15.6	4	3	0	5	3	1	6	4	2	6	6	3
22.0	2	3	4	4	5	7	5	6	8	7	7	8
30.8	4	3	4	6	7	5	8	8	6	9	9	7

* C_{TCM}, concentration of THI·CHI·Mix, mg/L

** R1, R2 and R3 represent for repeat 1, repeat 2 and repeat 3, respectively.
